# OsWOX3A is involved in negative feedback regulation of the gibberellic acid biosynthetic pathway in rice (*Oryza sativa*)

**DOI:** 10.1093/jxb/erv559

**Published:** 2016-01-14

**Authors:** Sung-Hwan Cho, Kiyoon Kang, Sang-Hwa Lee, In-Jung Lee, Nam-Chon Paek

**Affiliations:** ^1^Department of Plant Science, Plant Genomics and Breeding Institute, Research Institute of Agriculture and Life Sciences, Seoul National University, Seoul, 151–921, Republic of Korea; ^2^Division of Plant Biosciences, Kyungpook National University, Daegu, 702–701, Republic of Korea; ^3^Crop Biotechnology Institute, Institutes of Green Bio Science and Technology, Seoul National University, Pyeongchang, 232–916, Republic of Korea; ^4^Present address: Division of Plant Science and Biochemistry, University of Missouri, Columbia, MO 65211, USA

**Keywords:** Dwarfism, electrophoretic mobility shift assay, gibberellic acid, *KAO*, negative feedback regulation, *OsWOX3A*, rice.

## Abstract

The plant-specific WUSCHEL-related homeobox (WOX) nuclear proteins have important roles in the transcriptional regulation of many developmental processes. Among the rice (*Oryza sativa*) WOX proteins, a loss of OsWOX3A function in *narrow leaf2* (*nal2*) *nal3* double mutants (termed *nal2/3*) causes pleiotropic effects, such as narrow and curly leaves, opened spikelets, narrow grains, more tillers, and fewer lateral roots, but almost normal plant height. To examine OsWOX3A function in more detail, transgenic rice overexpressing *OsWOX3A* (*OsWOX3A*-OX) were generated; unexpectedly, all of them consistently exhibited severe dwarfism with very short and wide leaves, a phenotype that resembles that of gibberellic acid (GA)-deficient or GA-insensitive mutants. Exogenous GA_3_ treatment fully rescued the developmental defects of *OsWOX3A*-OX plants, suggesting that constitutive overexpression of *OsWOX3A* downregulates GA biosynthesis. Quantitative analysis of GA intermediates revealed significantly reduced levels of GA_20_ and bioactive GA_1_ in *OsWOX3A*-OX, possibly due to downregulation of the expression of *KAO*, which encodes *ent*-kaurenoic acid oxidase, a GA biosynthetic enzyme. Yeast one-hybrid and electrophoretic mobility shift assays revealed that OsWOX3A directly interacts with the *KAO* promoter. *OsWOX3A* expression is drastically and temporarily upregulated by GA_3_ and downregulated by paclobutrazol, a blocker of GA biosynthesis. These data indicate that *OsWOX3A* is a GA-responsive gene and functions in the negative feedback regulation of the GA biosynthetic pathway for GA homeostasis to maintain the threshold levels of endogenous GA intermediates throughout development.

## Highlight

The rice WUSCHEL-related homeobox transcription factor OsWOX3A affects homeostasis of gibberellic acid by functioning in the negative feedback regulation of gibberellic acid biosynthesis.

## Introduction

Semi-dwarfism is one of the most attractive and useful traits in cereal crop breeding because semi-dwarf plants show more resistance to lodging damage in unfavourable environments, such as wind and flood. Semi-dwarf plants also often show improved grain production owing to increased nitrogen-use efficiency (Borlaug, 1983; Evans, 1993; Khush, 1999; Hedden, 2003). Semi-dwarf variants of many crop plants have been identified and shown to enhance agronomic values. Indeed, at least 70 dwarf mutants have been reported in rice (*Oryza sativa*), and several of them have been characterized as gibberellic acid (GA)-deficient or GA-insensitive mutants (Matsuo, 1997; Itoh *et al.*, 2001; Sakamoto *et al.*, 2004; Asano *et al.*, 2009; Li *et al.*, 2011; Zhang *et al.*, 2014).

The essential phytohormone GA has pivotal roles in many developmental processes, such as seed germination, shoot and stem elongation, leaf expansion, flowering, and seed development (Achard and Genschik, 2009; Swain and Singh, 2005). In particular, GA is a major factor in determining plant height (Sakamoto and Matsuoka, 2004). GA metabolic pathways have been intensively analysed in plants (Hedden and Phillips, 2000; Olszewski *et al.*, 2002). GA intermediates are synthesized through several steps from geranylgeranyl diphosphate, which is converted to *ent*-kaurene by *ent*-copalyl diphosphate synthase (CPS) and *ent*-kaurene synthase (KS) (Aach *et al.*, 1997; Helliwell *et al.*, 2001). *ent*-Kaurene is thereafter converted to GA_12_ by two cytochrome P450 enzymes, *ent*-kaurene oxidase (KO) and *ent*-kaurenoic acid oxidase (KAO). Then, GA_12_ is converted to the bioactive GA_1_ form through the precursors GA_53_, GA_44_, GA_19_, and GA_20_ in the 13-hydroxylation pathway; GA_12_ is also converted to the bioactive GA_4_ form via GA_15_, GA_24_, and GA_9_ in the non-13-hydroxylation pathway (Sakamoto *et al.*, 2004; Yamaguchi, 2008). The overall rates of GA biosynthesis and deactivation maintain the levels of the bioactive forms of GA in plants (Hedden and Phillips, 2000). The flux of bioactive GA intermediates (i.e., GA_1_ and GA_4_) is regulated by three dioxygenases, GA 20-oxidase (GA20ox), GA 3-oxidase (GA3ox), and GA 2-oxidase (GA2ox). These enzymes have an important role in GA homeostasis (Hedden and Phillips, 2000). Both GA20ox and GA3ox catalyse the conversion of GA intermediates into bioactive forms, while GA2ox catalyses the conversion of bioactive GA intermediates into inactive catabolites (Yamaguchi, 2008).

In rice, several genes involved in GA metabolic pathways, including *D1*, *D18*, *D35*, *SD1*, *EUI*, and *BC12*/*GDD1*, affect plant height (Ueguchi-Tanaka *et al.*, 2000; Itoh *et al.*, 2001; Spielmeyer *et al.*, 2002; Itoh *et al.*, 2004; Zhu *et al.*, 2006; Li *et al.*, 2011). For example, loss-of-function mutations of *GA3ox2* (*d18*) and *GA20ox2* (*sd1*) resulted in dwarf plants (Sakamoto *et al.*, 2004). In particular, *GA3ox* and *GA20ox* are expressed in rapidly growing organs, including leaf primordia, young leaves, elongating internodes, and developing anthers and embryos (Kaneko *et al.*, 2003; Sakamoto *et al.*, 2004), whereas *CPS1*, *KS1*, *KO2*, *KAO*, and *GA2ox* are broadly expressed in many organs and tissues (Sakamoto *et al.*, 2004).

WUSCHEL (WUS)-related homeobox (WOX) nuclear proteins play key roles in coordinating transcription of many genes in various developmental processes (Haecker *et al.*, 2004). In particular, members of the *WOX3* subclade are involved in the regulation of lateral organ development. In *Arabidopsis*, *WOX3*/*PRESSED FLOWER* (*PRS*) is involved in the development of lateral-axis expansion of sepals and stipules (Matsumoto and Okada, 2001). Maize (*Zea mays*) WOX3 protein, encoded by the duplicated genes *NARROW SHEATH1* (*NS1*) and *NS2* (termed *NS1/2*), regulates shoot apical meristem and leaf development (Nardmann *et al.*, 2004). In rice, OsWOX3A protein, encoded by the duplicated genes *NARROW LEAF2* (*NAL2*) and *NAL3* (termed *NAL2/3*), plays important roles in lateral-axis outgrowth and vascular patterning in leaves and spikelets, development of tillers, and the formation of lateral roots and root hairs (Cho *et al.*, 2013; Yoo *et al.*, 2013). OsWOX3B protein, encoded by *DEPILOUS* (*DEP*), is required for trichome formation in leaves and glumes (Angeles-Shim *et al.*, 2012). Interestingly, transgenic rice plants overexpressing *OsWOX3A* (*OsWOX3A*-OX) exhibited a severe dwarf phenotype with wider leaves than wild type (Ishiwata *et al.*, 2013). Although recent work reported the possible functions of *WOX8*/*9* genes in the GA metabolic pathway (Wang *et al.*, 2014), the function of *WOX* genes in the GA metabolic pathway has not been fully elucidated.

This study showed that the severe dwarfism of *OsWOX3A*-OX plants was fully rescued by application of exogenous GA_3_. Quantification of endogenous GA intermediates revealed decreased levels of GA_20_ and GA_1_ in *OsWOX3A*-OX plants, because the expression of GA synthetic genes is altered by overexpression of *OsWOX3A*. Notably, OsWOX3A interacts directly with the *KAO* promoter to repress *KAO* expression. These results indicate that OsWOX3A is involved in the negative feedback regulation of GA biosynthesis for GA homeostasis throughout development in rice.

## Materials and methods

### Plant materials and growth conditions

The Korean *japonica* rice cultivar ‘Dongjinbye’ (hereafter termed wild type; WT) was used in this study. The full-length cDNA of *OsWOX3A* (accession no. AB218893) was isolated from WT. The rice mutant of *OsWOX3A*, *nal2*/*3*, was obtained from Kyushu University, as previously reported (Cho *et al.*, 2013). Plants were grown in the paddy field (Seoul National University Farm, Suwon, Korea) or in the growth chamber (12-h light at 30°C/12-h dark at 20°C).

### Vector construction and rice transformation

A 612-bp full-length *OsWOX3A* cDNA was amplified by reverse transcription PCR (RT-PCR) (primers listed in Supplementary Table S1). The cDNAs were cloned into pCR8/GW/TOPO (Invitrogen), followed by recombination into the binary vector pMDC32 (TAIR accession: 1009003741), a plant transformation vector containing a double cauliflower mosaic virus (CaMV) 35S promoter (Curtis and Grossniklaus, 2003). The recombinant plasmid was transformed into *Agrobacterium* strain EHA105 and introduced into the calli of mature embryos of WT (Jeon *et al.*, 2000). Transgenic plants developed from the calli were grown in Murashige and Skoog medium for 1 month, and confirmed by PCR with primers in the pMDC32 vector (35STC-F) and *OsWOX3A* fragment (TC-R) (Supplementary Table S1). To examine the expression levels of *OsWOX3A* in the transgenic rice plants, reverse transcription and quantitative real-time PCR (RT-qPCR) were conducted as previously described (Yoo *et al.*, 2009). The primers used for the *OsWOX3A* and *Ubiquitin5* (*Ub5*) (GenBank accession no. AK061988; Os01g0328400) genes are listed in Supplementary Table S1.

### Histochemical analysis of *OsWOX3A* expression

For β-glucuronidase (GUS) assays, transgenic rice plants containing the *ProOsWOX3A::GUS* transgene, which have previously been reported, were used (Cho *et al.*, 2013). GUS activity was detected histochemically as previously described (Jefferson *et al.*, 1987).

### Histological observation

To detect GUS activity in the elongating shoot in the *ProOsWOX3A::GUS* transgenic plants, 2-day-old seedlings were fixed in fixation solution (3.7% formaldehyde, 5% acetic acid, and 50% ethanol) overnight at 4°C, and dehydrated through a gradient series of ethanol, cleared in a xylene series, then infiltrated through a paraplast series (Sigma) for sections. The microtome sections (10–15 μm) were mounted on glass slides for imaging.

### Growth chemical treatments

The 2-week-old WT and *OsWOX3A*-OX seedlings were sprayed with 100ml of 10^–6^ M GA_3_ (in water) at 4–6h after dawn every day for 5 days. The length of the second leaf sheath was measured at 5 days of treatment. For *OsWOX3A* expression analysis, 2-week-old WT seedlings were sprayed with 50 μM GA_3_ or 10 μM paclobutrazol and harvested at different time points for RT-qPCR analysis. For expression analysis of *GA20ox2* and *GA3ox2*, WT and *OsWOX3A*-OX plants were sprayed with 50 μM GA_3_ and harvested after 3h for RT-qPCR analysis.

### RNA extraction and quantitative real-time PCR

For expression analysis of *OsWOX3A* and GA biosynthetic genes, the WT, *nal2*/*3* mutants (Cho *et al.*, 2014), and *OsWOX3A*-OX plants were grown in the growth chamber and leaf samples were harvested and homogenized in liquid nitrogen. Total RNA was extracted using an RNA extraction kit (RNeasy Plant Mini Kit, QIAGEN). Then, RT-qPCR was performed as previously described (Cho *et al.*, 2014). RT products equivalent to 50ng of total RNA and GoTaq qPCR Master Mix (Promega) were used in 50 μl reactions using the Light Cycler 480 (Roche). Roche Optical System software was used to calculate threshold cycle values. *Ub5* was used as an internal control. The relative expression of each gene was calculated using the 2^−∆∆*C*^_T_ methods as previously described (Livak and Schmittgen, 2001). The primers used for qPCR are listed in Supplementary Table S1.

### GA quantification

GA quantification was carried out as previously described (Foster and Morgan, 1995; Lee *et al.*, 1998). For accurate quantification, *OsWOX3A*-OX and *nal2*/*3* plants were planted on opposite sides of WT plants in the same pot to minimize environmental effects. The 4-week-old seedlings of *OsWOX3A*-OX, *nal2*/*3* mutants, and WT plants were harvested for quantitative GA analysis. After harvesting, the samples were immediately frozen in liquid nitrogen, freeze-dried, and ground into fine powder using a mortar and pestle. After extraction with methanol, GA intermediates were purified using a combination of preparatory column chromatography, solvent partitioning, and reverse-phase HPLC (Foster and Morgan, 1995). Deuterated internal standards were added (20ng each of [17,17−^2^H_2_]GA_1_, −GA_8_, −GA_19_, −GA_20_, −GA_29_, −GA_44_, and −GA_53_). GC-MS analysis was performed using a Hewlett-Packard model 6890 (Chemstation, USA). Gibberellin levels were calculated as the peak area ratios of endogenous (non-deuterated, sample) to deuterated GA intermediates, after correcting for any contribution from the deuterated standard to non-deuterated GA. The peak-area ratios of the following ion pairs, in the appropriate HPLC fractions and having a retention time similar to that of the corresponding GA intermediates, were determined to calculate the concentrations of endogenous GA intermediates by reference to calibration curves: 506/508 (GA_1_), 594/596 (GA_8_), 434/436 (GA_19_), 418/420 (GA_20_), 506/508 (GA_29_), 432/434 (GA_44_), and 448/450 (GA_53_).

### Yeast one-hybrid assay

The full-length coding sequence of *OsWOX3A* was amplified by PCR using the full-length cDNA (primers listed in Supplementary Table S1). The PCR product was inserted into the pGAD424 vector (Clontech) to fuse it with the GAL4 activation domain. To generate the reporter plasmid, fragments of the *CPS1*, *KO2*, *KAO*, *GA20ox2*, *GA3ox2*, *GA2ox1*, and *GA2ox3* promoters were amplified by PCR using genomic DNA with specific primers (Supplementary Table S1) that spanned from −1 to about −1000 in each promoter and PCR products were inserted into the pLacZi vectors (Clontech). These constructs were used to transform the yeast strain YM4571. All the procedures followed the manufacturer’s manual (Yeast Protocols Handbook PT3024–1; http://www.clontech.com/).

### Electrophoretic mobility shift assay

To produce the His-tagged OsWOX3A protein, the full-length *OsWOX3A* cDNA was inserted into the *Bam*HI and *Eco*RI sites of the expression vector pRSET-A (Invitrogen). The His-tagged construct was transformed into *Escherichia coli* BL21 (DE3). Cells were grown at 38°C and induced by the addition of isopropyl β-D-thiogalactopyranoside to a final concentration of 1mM when the OD_600_ of the culture was 0.4 to 0.6. The fusion protein was purified with Ni-NTA His-Bind Superflow beads (Novagen). Nucleotide sequences of the double-strand oligonucleotides for *KAO* used for EMSA are listed in Supplementary Table S1. The oligonucleotides were synthesized and labelled with biotin by Macrogen (Seoul, Korea). The DNA-binding reactions were performed at room temperature for 20min in 20 μl standard reaction mixtures [2mg purified proteins, 2 μl biotin-labelled annealed oligonucleotides, 2 μl of 10 × binding buffer (100mM Tris, 500mM KCl, and 10mM DTT, pH 7.5), 1 μl of 50% glycerol, 1 μl of 1% Nonidet P-40, 1 μl of 1M KCl, 1 μl of 100mM MgCl_2_, 1 μl of 200mM EDTA, 1 μl of 1mg/ml poly(deoxyguanylic-deoxycytidylic) acid, and 8 μl of water]. The samples were loaded onto 10% native polyacrylamide gel containing 45mM Tris, 45mM boric acid, and 1mM EDTA (TBE), pH 8.3. The gel was sandwiched and transferred to N^+^ nylon membrane (Millipore) in 0.5 × TBE buffer at 380 mA and 4°C for 1h. Biotin-labelled DNA was detected by the LightShift Chemi-luminescent EMSA kit (Pierce) following the manufacturer’s manual.

## Results

### *OsWOX3A* is expressed in rapidly growing organs

**Fig. 1.**
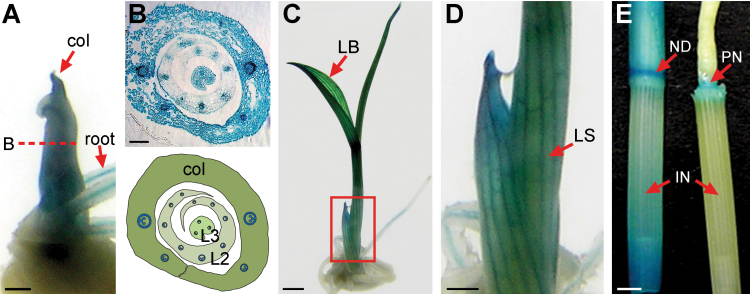
Expression pattern of *OsWOX3A*.

*OsWOX3A* is expressed in almost all tissues of rice plants, including leaf blades, leaf sheaths, and roots; in particular, it is highly expressed in the shoot base and the developing young panicles (Cho *et al.*, 2013). *In situ* hybridization experiments detected *OsWOX3A* transcripts in the vegetative shoots and in young leaves (Ishiwata *et al.*, 2013). Especially in the vegetative shoots, *OsWOX3A* transcripts were detected at the marginal edges of leaf primordia but not in the shoot apical meristem. In more detail, the *ProOsWOX3A::GUS* transgenic rice showed GUS expression in the coleoptile and vascular bundles of elongating shoots (Fig. 1A, B). The expression of *OsWOX3A* in young seedlings was detected in whole leaf blades and sheaths (Fig. 1C, D); GUS expression in leaf sheaths was mostly detected in vascular bundles and epidermal cells. Moreover, GUS expression was detected in elongating internodes, nodes, and panicle nodes (Fig. 1E). Therefore, *OsWOX3A* was broadly expressed in many different tissues, mostly in rapidly growing organs.

(**A, C**) Detection of GUS expression under the control of the *OsWOX3A* promoter (*ProOsWOX3A::GUS*) in 3-day-old elongating shoot in (A) and 1-week-old young seedling in (C). (**B**) Cross section of elongating shoot showing GUS expression in coleoptile, leaf primordia, and vasculature. (**D**) Magnified view of the bottom of leaf sheath in red rectangle in (C). (**E**) GUS expression in a 2-month-old node, elongating node (left) and young panicle node (right). col, coleoptile; IN, internode; L2, 2nd leaf; L3, 3rd leaf; LB, leaf blade; LS, leaf sheath; ND, node; PN, panicle node;. Scale bars = 1mm (A), 0.2mm (B), 4mm (C), 2mm (D, E). These experiments were repeated more than twice with similar results.

### Overexpression of *OsWOX3A* resulted in a severe dwarf phenotype

**Fig. 2.**
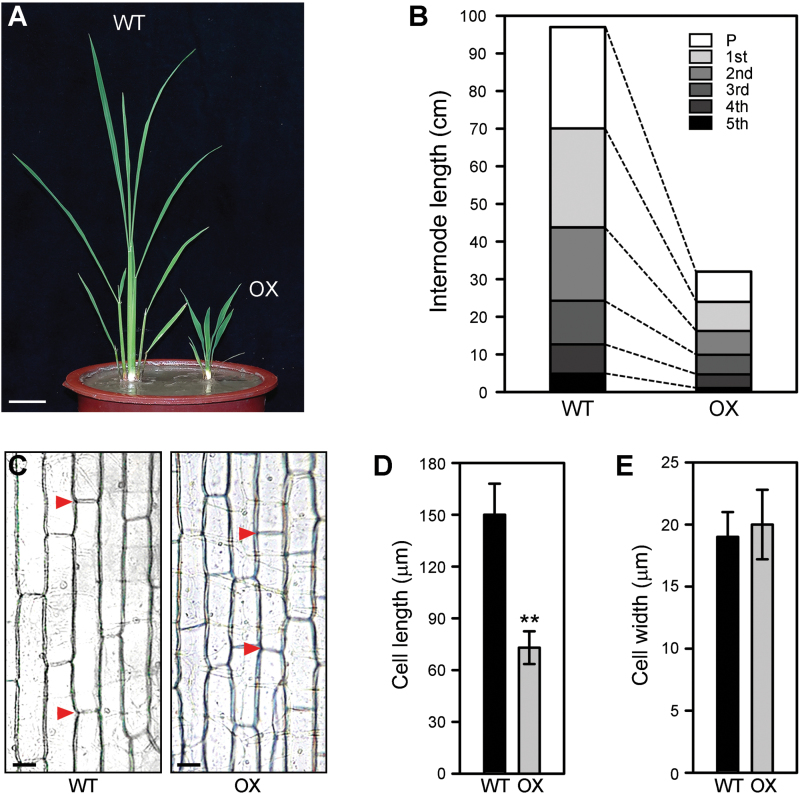
*OsWOX3A*-OX induces severe dwarfism.

To study the function of *OsWOX3A*, *OsWOX3A*-OX plants constitutively expressing *OsWOX3A* under the control of the double *CaMV35S* promoters were generated. To this end, the *Pro35S*(2x)*::OsWOX3A* construct was introduced into the calli derived from the mature embryos of *japonica* WT cultivar ‘Dongjinbyeo’ by *Agrobacterium*-mediated transformation and seven independent T_0_ transgenic plants were obtained from the transgenic calli (Supplementary Fig. S1). All transgenic plants displayed a severe dwarf phenotype with dark green and much wider leaf blade compared with WT (Fig. 2A; Supplementary Fig. S1A–C). Ishiwata *et al.* (2013) reported that the *ProACTIN1::OsWOX3A* transgenic rice showed a very similar phenotype to the *Pro35S*(2×)*::OsWOX3A* transgenic rice plants produced here. The height of *OsWOX3A*-OX plants was approximately one-quarter that of WT (Fig. 2A; Supplementary Fig. S1A). After heading, the panicle and internodes were shorter than WT (Fig. 2B). In addition, the epidermal cells of the second leaf sheath were much shorter (Fig. 2C), indicating that cell elongation becomes markedly reduced in *OsWOX3A*-OX plants (Fig. 2C, D). However, the width of epidermal cells in the second leaf sheath was not altered (Fig. 2E). *OsWOX3A*-OX panicles were much shorter than WT and thus the panicles had many fewer spikelets; no alteration of spikelet shape was observed, but grains of the transgenic plants were slightly shorter than WT (Supplementary Fig. S2A). In addition, the *OsWOX3A*-OX plants had more lateral roots but slightly fewer adventitious roots than WT (Supplementary Fig. S2B). The number of lateral roots in the same region of the primary root did not significantly differ between WT and *OsWOX3A*-OX (Supplementary Fig. S2B). Interestingly, the *OsWOX3A*-OX lateral roots were considerably shorter than WT lateral roots (Supplementary Fig. S2C). Thus, in addition to its previously reported functions (Cho *et al.*, 2013; Yoo *et al.*, 2013), OsWOX3A also can function in the inhibition of the longitudinal elongation of cells in both vegetative and reproductive organs during rice development.

(**A**) The 1-month-old plants grown in the paddy field. WT, wild type. OX, *OsWOX3A*-OX. **(B)** Internode lengths of plants at 2 weeks after heading. The average values were calculated from measurement of at least 15 plants. P, panicle; 1st to 5th nodes, respectively. (C-E) Length of epidermal cells of second leaf sheath in 1-month-old plants. (**C**) Red arrows indicate the upper or lower limits of epidermal cells. (**D, E**) Quantitative measurement of the cell lengths (D) and widths (E) of second leaf sheath. Data are mean ± SD from at least 15 plants. Significant difference was determined by Student’s *t*-test (**P < 0.01). Scale bars = 4cm (A), 25mm (C). These experiments were repeated more than twice with similar results.

### Exogenous GA_3_ treatment rescued the dwarfism phenotype of *OsWOX3A*-OX plants

**Fig. 3.**
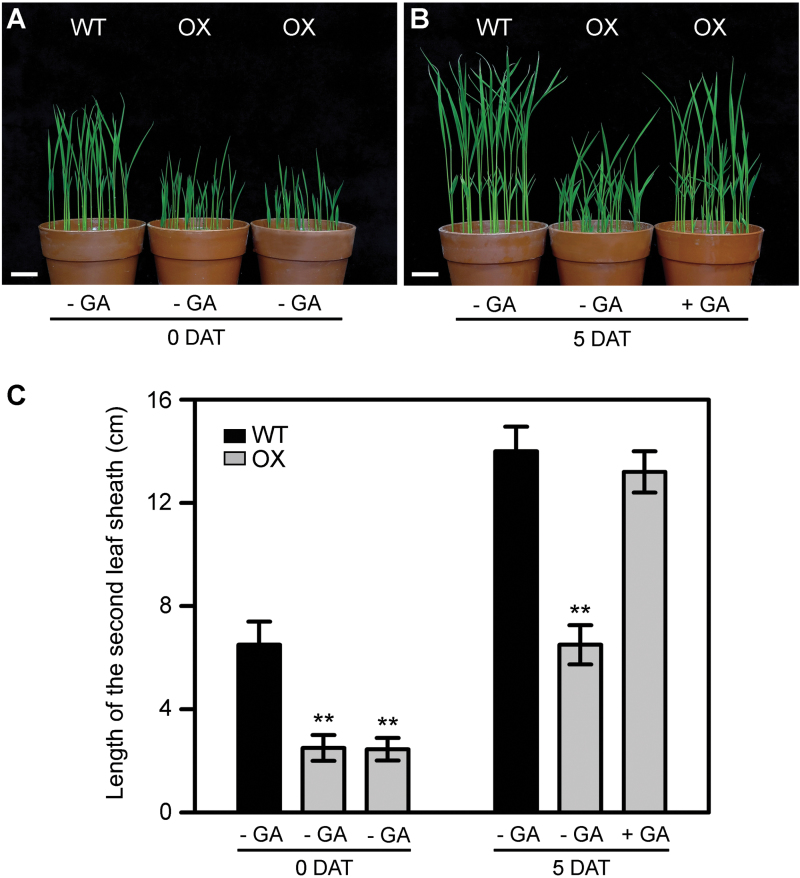
Rescue of dwarf phenotype by exogenous GA_3_ treatment.

GA, the most important hormone regulating the longitudinal growth and elongation of plant cells, plays a major role in determining plant height (Sakamoto and Matsuoka, 2004). To identify whether the severe dwarf phenotype of *OsWOX3A*-OX plants was caused by GA deficiency or GA insensitivity, the 2-week-old *OsWOX3A*-OX plants were treated with GA_3_ by spraying with 10^–6^ M or 10^–8^ M GA_3_ for 5 days and measuring their heights. The lengths of the second leaf sheath of both WT and *OsWOX3A*-OX plants did not change in response to treatment with 10^–8^ M GA_3_ (data not shown). In response to treatment with 10^–6^ M GA_3_, the sheath length of *OsWOX3A*-OX was fully rescued, becoming similar to WT (Fig. 3). This result suggests that *OsWOX3A*-OX plants are possibly deficient in bioactive GA. This observation is consistent with the GA-deficient cells that exhibit impairment or retardation of cell elongation throughout development in rice (Dai *et al.*, 2007b; Li *et al.*, 2011).

(**A, B**) Plants were grown in soil for 14 days (A) and then sprayed with 10^–6^ M GA_3_ in 100ml water and photographed after 5 days. DAT, day(s) after treatment; OX, *OsWOX3A*-OX. The lengths of the second leaf sheath are rescued in the *OsWOX3A*-OX plants (right) in B. (**C**) Quantitative measurement of the length of the second leaf sheath. Data are mean ± SD from at least 15 plants. Significant differences compared with WT were determined by Student’s *t*-test (**P < 0.01). Scale bars = 2cm (A, B). These experiments were repeated more than twice with similar results.

### Endogenous levels of GA in *OsWOX3A*-OX plants

**Fig. 4.**
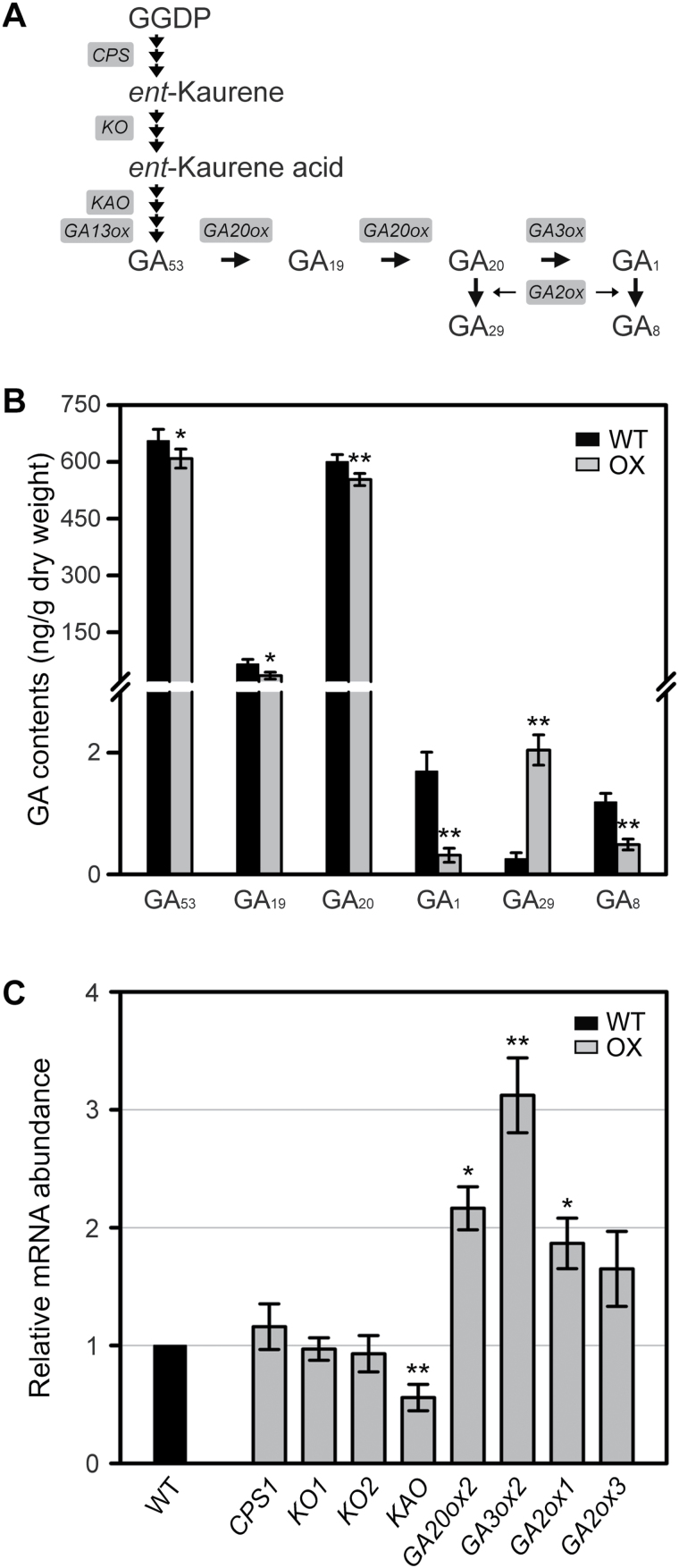
*OsWOX3A*-OX altered GA accumulation and expression levels of GA metabolic genes.

GA_1_ is the major bioactive GA that regulates longitudinal elongation of vegetative organs in rice (Kobayashi, 1988). To determine endogenous levels of GA, the levels of different 13-hydroxylated GA intermediates within the GA_1_ metabolic pathway were measured (Fig. 4A) using GC-MS. In *OsWOX3A*-OX plants, the bioactive GA_1_ level decreased to about 20% of the WT level. Furthermore, the levels of GA_20_ (precursor of GA_1_), GA_8_ (deactivated form of GA_1_), GA_53_ (upstream precursor), and GA_19_ (upstream precursor) were significantly lower, whereas the level of GA_29_ (deactivated form of GA_20_) was about 8-fold higher in *OsWOX3A*-OX plants compared with WT (Fig. 4B). This result strongly suggests that ectopic and constitutive overexpression of *OsWOX3A* negatively affects the GA biosynthetic pathway throughout development.

(**A**) Schematic representation of GA biosynthetic genes. (**B**) Quantification of 13-hydroxylated GA intermediates in 4-week-old seedlings. GA_12_ had undetectably low abundance. Data are means ± SD from three biological samples (ng/g dry weight). Significant difference was determined by Student’s *t*-test (*P < 0.05, **P < 0.01). (**C**) RT-qPCR analysis of eight genes in the GA metabolic pathway in 4-week-old *OsWOX3A-OX* plants. Relative mRNA levels of each gene were normalized to the mRNA levels of *Ub5* (Os01g0328400). Expression levels for each gene in *OsWOX3A*-OX are shown relative to the expression in WT, which is set as 1. Data are means ± SD from three biological repeats. Significant difference was determined by Student’s *t*-test (*P < 0.05, **P < 0.01). These experiments were repeated more than twice with similar results.

### *OsWOX3A*-OX alters the expression of GA biosynthetic genes

Changes of the GA intermediate levels in *OsWOX3A*-OX plants may be caused by altered expression of genes encoding GA metabolic enzymes. Thus, the expression levels of GA biosynthesis genes, such as *CPS*, *KO1*, *KO2*, *KAO*, *GA20ox2*, *GA3ox2*, *GA2ox1*, and *GA2ox3* were compared between WT and *OsWOX3A*-OX plants (Fig. 4C). Interestingly, the expression of *KAO*, whose product catalyses the oxidation of *ent*-kaurenoic acid, was drastically downregulated in *OsWOX3A*-OX plants (Fig. 4C). However, the expression of *CPS1*, *KO1*, and *KO2* showed no significant difference between WT and *OsWOX3A*-OX plants. Unusually, in *OsWOX3A*-OX plants, expression levels of *GA20ox2*, *GA3ox2*, *GA2ox1*, and *GA2ox3* were upregulated to about 2–3-fold higher than in WT. *GA20ox2* and *GA3ox2* are the major negative feedback regulators that maintain the threshold levels of endogenous GA (Itoh *et al.*, 2001; Itoh *et al.*, 2002). To further understand the upregulation of *GA20ox2* and *GA3ox2* expression in *OsWOX3A*-OX plants, the expression patterns of these genes were compared between GA_3_-treated WT and *OsWOX3A*-OX plants. The exogenous GA_3_ greatly downregulated their expression in both WT and *OsWOX3A*-OX plants (Supplementary Fig. S3), demonstrating that GA deficiency causes the dwarfism of *OsWOX3A*-OX plants (Fig. 4A).

### Alteration of GA biosynthetic gene expression and endogenous GA levels in *nal2/3* mutants

**Fig. 5.**
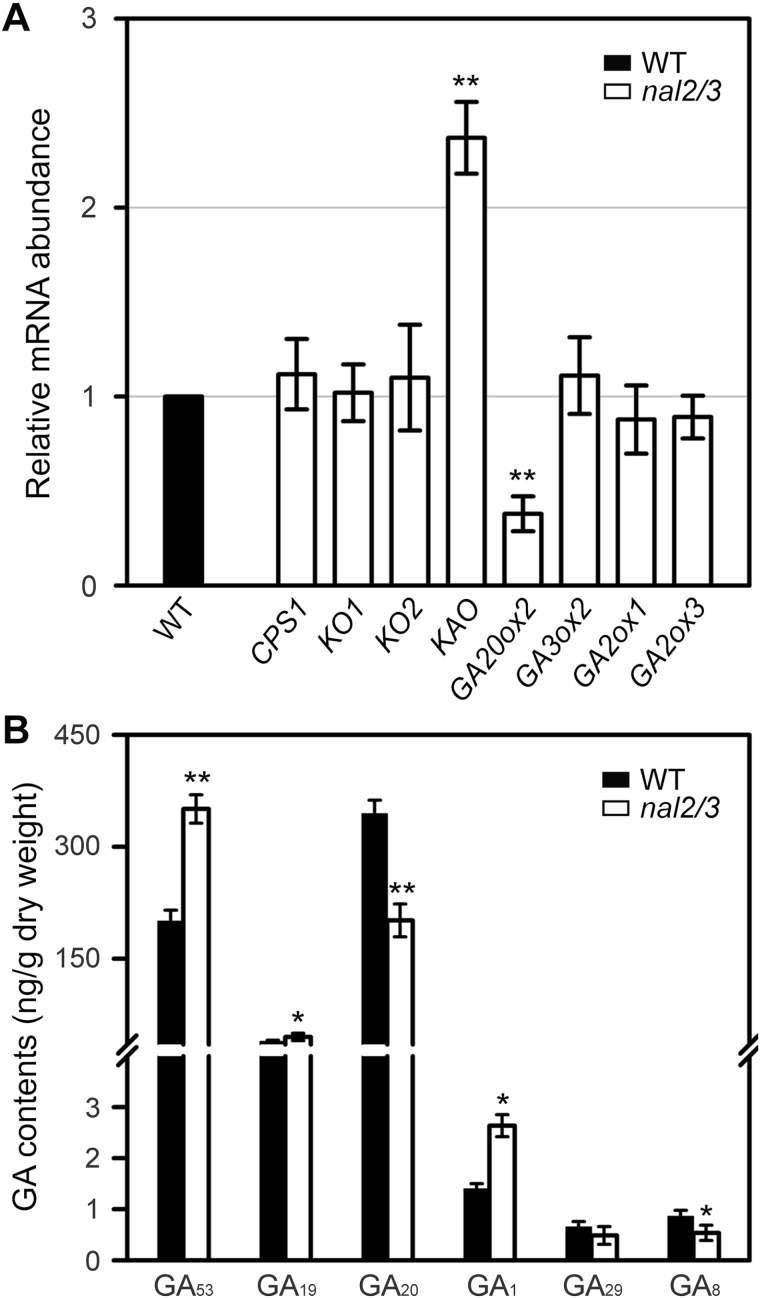
Loss of *OsWOX3A* activity altered GA accumulation and expression of GA metabolic genes.

To further investigate the function of OsWOX3A in the GA biosynthetic pathway, the expression levels of GA biosynthetic genes were examined in *nal2/3* mutants (Cho *et al.*, 2013). RT-qPCR showed about a 2-fold increase in *KAO* expression compared with WT (Fig. 5A). The expression of *GA20ox2* was downregulated in *nal2/3* mutants. However, the expression levels of *GA3ox2*, *GA2ox1*, and *GA2ox3* were not altered. Analysis of 13-hydroxylated GA intermediates in *nal2/3* mutants showed significant increases in GA_53_ and GA_19_ levels, and decreases in GA_20_ and GA_8_ levels, compared with WT (Fig. 5B). Overall, bioactive GA_1_ slightly increased in *nal2/3* mutants (Fig. 5B). The changes in GA contents are consistent with the expression levels of *KAO* and *GA20ox2* in *nal2/3* mutants. The increased expression of *GA20ox2*, *GA3ox2*, *GA2ox1*, and *GA2ox3* in *OsWOX3A*-OX plants (Fig. 4B) and decreased expression of *GA20ox2* (Fig. 5A) in *nal2*/*3* mutants might be achieved by indirect mechanisms (e.g. altered auxin distribution). Thus, it can be speculated that *OsWOX3A* directly downregulates the expression of *KAO* as a *trans*-repressor or upregulates the expression of *GA20ox2* as a *trans*-activator.

(**A**) RT-qPCR analysis of the eight genes in the GA metabolic pathway in 4-week-old *nal2*/*3* mutants. Relative mRNA levels of each gene are normalized to the mRNA levels of *Ub5* (Os01g0328400). Expression levels for each gene in *nal2/3* mutants are shown relative to the expression in WT, which is set as 1. Data are means ± SD from three biological repeats. Significant difference was determined by Student’s *t*-test (**P < 0.01). (**B**) Quantification of 13-hydroxylated GA intermediates in 4-week-old seedlings. GA_12_ had undetectably low abundance. Data are means ± SD from three biological repeats (ng/g dry weight). Significant difference was determined by Student’s *t*-test (*P < 0.05, **P < 0.01). These experiments were repeated more than twice with similar results.

### OsWOX3A protein interacts with the *KAO* promoter to repress its expression

**Fig. 6.**
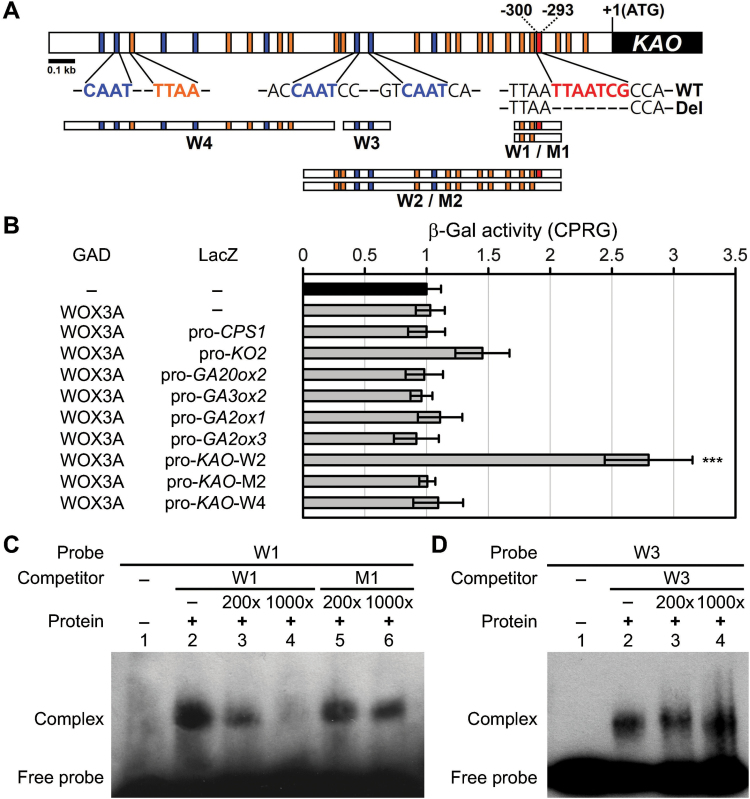
OsWOX3A protein directly binds to the promoter region of *KAO*.

To test whether the OsWOX3A protein directly interacts with the promoters of *KAO* or *GA20ox2*, yeast one-hybrid assays were used to test the promoter regions of *KAO*, *GA20ox2*, and other genes in the GA synthetic pathway. The assays showed that OsWOX3A only binds to the ~1kb promoter region of *KAO* (W2), but not to the promoters of *GA20ox2* or other tested genes (Fig. 6B). The W4 promoter region from −2kb to −1kb of *KAO* was also tested by yeast one-hybrid assay, which showed that OsWOX3A does not bind to this region of *KAO* (Fig. 6A, B).

(**A**) The locations of the W1, M1, W2, M2, W3, and W4 probes within the promoter region of *KAO*. The W1 probe includes the TTAATCG sequence, which is shown in red letters and by a red box. Reported core binding elements for WUS are indicated in blue and orange and by blue and orange boxes. (**B**) Analysis of OsWOX3A binding to the promoters of GA metabolic genes (*CPS1*, *KO2*, *KAO*, *GA20ox2*, *GA3ox2*, *GA2ox1*, and *GA2ox3*) using yeast one-hybrid assays. β-Galactosidase (β-Gal) activity was measured by liquid assay using chlorophenol red-β-D-galactopyranoside (CPRG). Each promoter binding activity is shown relative to the CPRG unit (10^4^ ml^−1^ min^−1^) of the negative control that contains empty bait and prey plasmids (–), which is set as 1. Data are means ± SD from six independent colonies. Significant difference was determined by Student’s *t*-test (***P < 0.001). (**C, D**) EMSA showing His-OsWOX3A fusion protein binding to the *KAO* promoter. Oligonucleotides containing W1 (the *KAO* promoter binding site) or W3 (the *KAO* promoter with reported binding element for WUS), were used as the biotin-labelled probes. The W1 probe is shown in red letters in (A) and the M1 probe is shown with a dotted line in Del in (A). The negative control is indicated in Lane 1 in (C, D). Biotin-unlabelled W1, M1 (deleted version of W1), and W3 were used as the unlabelled competitors. The (+) presence or (−) absence of His-OsWOX3A fusion protein is indicated. These experiments were repeated more than three times with similar results.

To see whether the promoter of *KAO* contains reported target motifs for WOX binding, 2kb of sequence in the *KAO* promoter region was examined. This sequence analysis revealed that the *KAO* promoter region has consensus sequences for the motifs CAAT (eight occurrences), TTAA (19 occurrences), and TTAATCG (one occurrence) (Fig. 6; Supplementary Fig. S4), which have been reported as target binding motifs for WOX proteins (Lohmann *et al.*, 2001; Busch *et al.*, 2010; Franco-Zorrilla *et al.*, 2014). In spite of the many CAAT and TTAA sequences, OsWOX3A failed to bind to the W4 promoter region. To examine the importance of the TTAATCG motif, a construct with a deleted TTAATCG sequence (M2) was tested with a yeast one-hybrid assay, which showed that OsWOX3A does not bind to the deleted M2 probe from the promoter of *KAO* (Fig. 6B). These results suggest that OsWOX3A may bind to the TTAATCG motif in the promoter of *KAO*. To confirm if binding of OsWOX3A requires the consensus sequence of the *KAO* promoter, EMSA was carried out using the His-fusion OsWOX3A (His-OsWOX3A) produced in *E. coli* (Supplementary Fig. S5). The consensus binding sequence of *KAO* (W1) and deleted sequence lacking the TTAATCG (M1) were used as probes (Fig. 6A). The EMSA revealed that the His-OsWOX3A protein did bind to the consensus W1 sequence but not to the M1 sequence (Fig. 6C); in addition, OsWOX3A failed to interact with the repeated CAAT motif (W2) (Fig. 6D). Taken together, these results indicate a direct involvement of OsWOX3A in downregulating the expression of *KAO*.

### Exogenous GA upregulates *OsWOX3A* expression

**Fig. 7.**
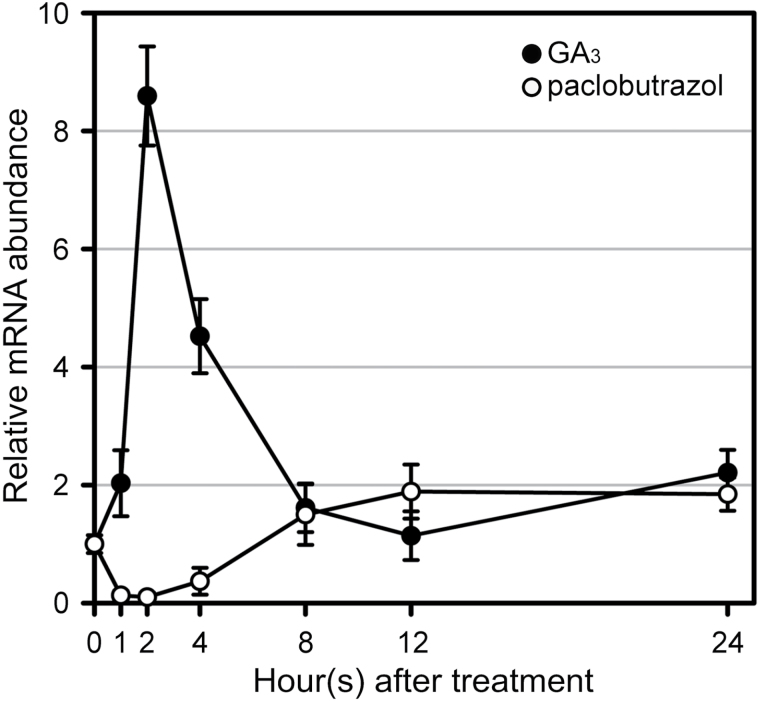
Transient, rapid alteration of *OsWOX3A* expression by GA_3_ and paclobutrazol.

The expression patterns of *OsWOX3A* in shoot base or elongating stem were quite similar to those of GA biosynthetic genes (Fig. 1; Cho *et al.*, 2013; Kaneko *et al.*, 2003). Thus, this study tested whether exogenous GA_3_ treatment alters the expression of *OsWOX3A*. To this end, 2-week-old WT seedlings were sprayed once with 50 μM GA_3_, and then the aerial parts were harvested at 0 to 24h. RT-qPCR analysis showed that *OsWOX3A* expression rapidly increased almost 9-fold at 2h after treatment and then decreased to control levels at 8h after treatment (Fig. 7). Furthermore, the effect of a well-known inhibitor of GA biosynthesis, paclobutrazol, on *OsWOX3A* expression was examined. After treatment with10 μM paclobutrazol, 2-week-old WT plants were harvested at the same time points as for the GA_3_ treatment. Application of paclobutrazol and GA_3_ treatment caused opposite changes in the expression of *OsWOX3A* (Fig. 7). These observations strongly suggest that the temporal increase of endogenous GA levels rapidly induces the expression of *OsWOX3A*, possibly decreasing the rate of GA biosynthesis and affecting GA homeostasis.

RT-qPCR analysis of *OsWOX3A* mRNA levels in 4-week-old WT seedlings treated with 50 μM GA_3_ or 10 μM paclobutrazol. Relative mRNA levels normalized to the mRNA levels of *Ub5* (Os01g0328400). Expression levels of *OsWOX3A* are relative to the expression at time point zero (control; no treatment), which is set as 1. Data are means ± SD from three biological repeats. These experiments were repeated more than twice with similar results.

## Discussion

The phenotypic and molecular genetic analysis of *nal2/3* mutants in rice demonstrated that *OsWOX3A* has a conserved role similar to those of *NS1/2* of maize and *PRS* of *Arabidopsis* in the regulation of lateral-axis outgrowth and margin development in founder cells and lateral organ primordia (Nardmann *et al.*, 2004; Cho *et al.*, 2013; Ishiwata *et al.*, 2013). Interestingly, unlike *ns1/2* and *prs* mutants, mutation of *WOX3A* has a pleiotropic effect in rice (Cho *et al.*, 2013). However, other functions of *OsWOX3A* throughout development have remained unknown. The spatial expression of *OsWOX3A* overlaps with that of the GA biosynthetic genes, which are expressed in several organs, including vegetative shoot base, leaf sheaths, leaf blades, and elongating stems (Fig. 1; Sakamoto *et al.*, 2004; Cho *et al.*, 2013). This study provides evidence that OsWOX3A functions in the negative feedback regulation of the GA biosynthetic pathway for GA homeostasis throughout development.

### OsWOX3A has a negative role in GA biosynthesis at the transcriptional level

To date, only one study has reported a regulatory role of a WOX protein in GA signalling; *DWARF TILLER1*, a homolog of *Arabidopsis WOX8* or *WOX9*, is required for tiller and shoot growth through GA signalling (Wang *et al.*, 2014). The rice mutant of *KAO* (*oskao-1*) has low levels of GA_53_, GA_20_, and GA_1_ (Sakamoto *et al.*, 2004). This study showed that *OsWOX3A*-OX downregulates *KAO* expression, which causes significantly reduced levels of GA_53_, GA_20_, and, finally, bioactive GA_1_ (Fig. 4). Loss-of-function *nal2/3* mutants showed increased expression of *KAO* (Fig. 5). Notably, OsWOX3A interacts with a WOX-binding motif, TTAATCG, in the *KAO* promoter (Fig. 6). These results suggest that OsWOX3A is involved in the negative feedback regulation of *KAO* expression for GA homeostasis.

GA biosynthesis is controlled by negative feedback regulation through bioactive GA intermediates, and *GA20ox2* and *GA3ox2* act as major negative feedback regulators in rice (Itoh *et al.*, 2001; Itoh *et al.*, 2002). Exogenous GA_3_ treatment of WT and *OsWOX3A*-OX plants markedly downregulated the expression of both *GA20ox2* and *GA3ox2* (Supplementary Fig. S3). Therefore, upregulation of *GA20ox2* and *GA3ox2* expression in *OsWOX3A*-OX plants might be caused by negative feedback regulation under low levels of bioactive GA_1_. However, *nal2/3* mutants did not show decreased expression of *GA3ox2* (Fig. 5), which helps explain why the *nal2/3* mutants showed a slight increase of GA_1_ accumulation (Fig. 5). Interestingly, *nal2*/*3* mutants showed decreased *GA20ox2* expression, which might be associated with lower levels of GA_20_ (Fig. 5). In this scenario, a reduction of *GA20ox2* expression in the *nal2*/*3* mutants might be caused by negative feedback regulation in response to the increase of bioactive GA_1_. In addition, the promoter region of *GA20ox2* does not have an OsWOX3A binding motif, strongly suggesting that *OsWOX3A* is indirectly involved in *GA20ox2* expression. Likewise, *OsWOX3A* indirectly upregulates *GA2ox3* or *GA2ox1*, consistent with higher accumulation of GA_29_ from the GA_20_ intermediate in *OsWOX3A*-OX plants, whereas these plants have reduced levels of GA_8_ caused by low levels of GA_1_ (Fig. 4). The *nal2/3* mutation did not affect the expression of *GA2ox* genes, probably owing to a slight increase of GA_1_ levels. Moreover, reduced expression of *GA20ox2* and a slight accumulation of more bioactive GA_1_ (Fig. 5) did not increase the height of *nal2/3* mutant plants (Cho *et al.*, 2013). Interestingly, it has also been reported that auxin controls the expression of GA metabolic genes and altered auxin distribution plays a role in regulating the GA biosynthetic genes (Desgagne-Penix and Sponsel, 2008; Frigerio *et al.*, 2006). Thus, the increase of GA_1_ in *nal2*/*3* mutants may not be sufficient to affect cell elongation.

Previous work reported that *OsWOX3A* is involved in the formation of lateral roots, possibly by regulating auxin-related genes (Cho *et al.* 2013). GA intermediates negatively affect lateral root formation by inhibiting lateral root primordium initiation via modification of polar auxin transport (Gou *et al.*, 2010). Therefore, although more physiological and biochemical studies are needed, the expression pattern of *OsWOX3A* in roots and the alteration of formation and elongation in the lateral roots in *OsWOX3A*-OX plants suggest that *OsWOX3A* may function in roots through the crosstalk between GA-related and auxin-related pathways (Fig. 1A; Supplementary Fig. S2B, C).

### OsWOX3A is involved in negative feedback regulation of the GA biosynthetic pathway for GA homeostasis

In plants, the balance between GA biosynthesis and degradation tightly controls the levels of bioactive GA intermediates. In particular, GA biosynthesis is controlled by feedback regulation through the activity of the GA-responsive pathway, because plant growth and development require precise GA homeostasis. The expression of *OsWOX3A* is GA-responsive, because it is rapidly but temporarily activated by exogenous GA treatment and suppressed by the GA biosynthesis inhibitor paclobutrazol (Fig. 7). This suggests that, at least in part, *OsWOX3A* might be involved in the regulation of a feedback pathway in GA biosynthesis. OsWOX3A binds to the promoter of *KAO*, and thus may be closely associated with the GA activity-dependent downregulation of *KAO* expression (Fig. 6; Supplementary Fig. S4, S5). In this model, rapid activation of *OsWOX3A* by GA suppresses the expression of *KAO* and consequently decreases endogenous levels of bioactive GA intermediates, which later leads to downregulation of *OsWOX3A* expression.

### OsWOX3A acts as a transcriptional repressor

The target sequence of WUS, TTAAT(G/C)(G/C), occurs in the intron of *AGAMOUS* in *Arabidopsis* (Lohmann *et al.*, 2001). Similarly, rice QHB, WOX3, and WOX11 proteins also bind to the TTAATGG sequence (Kamiya *et al.*, 2003; Dai *et al.*, 2007a; Zhao *et al.*, 2009). In addition, WUS protein specifically recognized the sequence CACGTG (Busch *et al.*, 2010), and two binding core sequences for WOX13 (CAAT and TTAA) have been identified (Franco-Zorrilla *et al.*, 2014). These studies suggest that CACGTG, CAAT, TTAA, and TTAAT(G/C)(G/C) are the consensus sequences for WUS-binding and WOX-binding motifs.

Here, analysis of the promoters of GA biosynthetic genes revealed that only the *KAO* promoter (~2kb) contains a WOX-binding motif (Fig. 6A; Supplementary Fig. S4). Yeast one-hybrid assays and EMSA supported the idea that OsWOX3A can interact with the WOX-binding motif of the *KAO* promoter (Fig. 6; Supplementary Fig. S4–S6). A previous study reported that OsWOX3A acts as a transcriptional repressor of *YABBY3* during leaf development (Dai *et al.*, 2007a). However, in contrast to its role as a repressor, it has been reported that OsWOX3A may act as a transcriptional activator of leaf development and auxin-related genes (Cho *et al.*, 2013; Ishiwata *et al.*, 2013). Ikeda *et al.* (2009) found that WUS is a bifunctional transcriptional factor that acts as a repressor but also acts as a direct activator of the expression of the *AGAMOUS* gene. Taken together, these results indicate that OsWOX3A might act as a transcriptional repressor rather than an activator in the GA pathway. Furthermore, our physiological study revealed that severe dwarfism of *OsWOX3A*-OX plants can be rescued by exogenous GA_3_ treatment (Fig. 2). Taking these observations together, this study indicates that OsWOX3A is involved in the negative feedback regulation of GA homeostasis during growth and development in rice.

## Supplementary data

Supplementary data are available at *JXB* online.

Fig. S1. Phenotypic characteristics of *OsWOX3A*-OX (OX) plants.

Fig. S2. Multiple developmental defects in *OsWOX3A*-OX (OX) plants.

Fig. S3. Effect of exogenous GA_3_ treatment on the relative expression of *GA20ox2* and *GA3ox2* in 4-week-old wild type (WT) and *OsWOX3A*-OX (OX) plants.

Fig. S4. Analysis of OsWOX3A-binding motifs in the promoter of *KAO*.

Fig. S5. Expression of recombinant OsWOX3A fusion protein in *E. coil*.

Fig. S6. OsWOX3A does not bind to the M1 promoter region of *KAO*.

Table S1. Primers used in this study.

Supplementary Data

## References

[CIT0001] Aach H Bode H Robinson DG Graebe JE 1997 *ent*-Kaurene synthase is located in proplastids of meristematic shoot tissues. Planta 202, 211–219.

[CIT0002] Achard P Genschik P 2009 Releasing the brakes of plant growth: how GAs shutdown DELLA proteins. Journal of Experimental Botany 60, 1085–1092. 19043067 10.1093/jxb/ern301

[CIT0003] Angeles-Shim R Asano K Takashi T Shim J Kuroha T Ayano M Ashikari M 2012 A WUSCHEL-related homeobox 3B gene, *depilous (dep*), confers glabrousness of rice leaves and glumes. Rice 5, 28. 10.1186/1939-8433-5-28 PMC5520829 27234246

[CIT0004] Asano K Hirano K Ueguchi-Tanaka M 2009 Isolation and characterization of dominant dwarf mutants, *Slr1-d*, in rice. Molecular Genetics and Genomics 281, 223–231. 19066966 10.1007/s00438-008-0406-6

[CIT0005] Borlaug NE 1983 Contributions of conventional plant breeding to food production. Science 219, 689–693. 17814030 10.1126/science.219.4585.689

[CIT0006] Busch W Miotk A Ariel FD 2010 Transcriptional control of a plant stem cell niche. Developmental Cell 18, 849–861. 20493817 10.1016/j.devcel.2010.03.012

[CIT0007] Cho S-H Yoo S-C Zhang H Lim J-H Paek N-C 2014 Rice NARROW LEAF1 regulates leaf and adventitious root development. Plant Molecular Biology Reporter 32, 270–281.

[CIT0008] Cho S-H Yoo S-C Zhang H Pandeya D Koh H-J Hwang J-Y Kim G-T Paek N-C 2013 The rice *narrow leaf2 and narrow leaf3* loci encode WUSCHEL-related homeobox 3A (OsWOX3A) and function in leaf, spikelet, tiller and lateral root development. New Phytologist 198, 1071–1084. 23551229 10.1111/nph.12231

[CIT0009] Curtis MD Grossniklaus U 2003 A gateway cloning vector set for high-throughput functional analysis of genes *in planta* . Plant Physiology 133, 462–469. 14555774 10.1104/pp.103.027979 PMC523872

[CIT0010] Dai M Hu Y Zhao Y Liu H Zhou DX 2007a A *WUSCHEL-LIKE HOMEOBOX* gene represses a *YABBY* gene expression required for rice leaf development. Plant Physiology 144, 380–390. 17351053 10.1104/pp.107.095737 PMC1913789

[CIT0011] Dai M Zhao Y Ma Q Hu Y Hedden P Zhang Q Zhou DX 2007b The rice YABBY1 gene is involved in the feedback regulation of gibberellin metabolism. Plant Physiology 144, 121–133. 17369428 10.1104/pp.107.096586 PMC1913802

[CIT0012] Desgagne-Penix I Sponsel VM 2008 Expression of *gibberellin 20-oxidase1* (*AtGA20ox1*) in *Arabidopsis* seedlings with altered auxin status is regulated at multiple levels. Journal of Experimental Botany 59, 2057–2070. 18440929 10.1093/jxb/ern063 PMC2413289

[CIT0013] Evans LT 1993 Adaptation and the ecology of yield. In: Evans LT ed. Crop evolution, adaptation and yield . Cambridge: Cambridge University Press p. 113–168.

[CIT0014] Foster KR Morgan PW 1995 Genetic regulation of development in *Sorghum bicolor* (IX. The ma3R allele disrupts diurnal control of gibberellin biosynthesis). Plant Physiology 108, 337–343. 12228478 10.1104/pp.108.1.337 PMC157339

[CIT0015] Franco-Zorrilla JM Lopez-Vidriero I Carrasco JL Godoy M Vera P Solano R 2014 DNA-binding specificities of plant transcription factors and their potential to define target genes. Proceedings of the National Academy of Sciences, USA 111, 2367–2372. 10.1073/pnas.1316278111 PMC3926073 24477691

[CIT0016] Frigerio M Alabadi D Perez-Gomez J Garcia-Carcel L Phillips AL Hedden P Blazquez MA 2006 Transcriptional regulation of gibberellin metabolism genes by auxin signaling in Arabidopsis. Plant Physiology 142, 553–563. 16905669 10.1104/pp.106.084871 PMC1586059

[CIT0017] Gou J Strauss SH Tsai CJ Fang K Chen Y Jiang X Busov VB 2010 Gibberellins regulate lateral root formation in *Populus* through interactions with auxin and other hormones. The Plant Cell 22, 623–639. 20354195 10.1105/tpc.109.073239 PMC2861444

[CIT0018] Haecker A Gross-Hardt R Geiges B Sarkar A Breuninger H Herrmann M Laux T 2004 Expression dynamics of *WOX* genes mark cell fate decisions during early embryonic patterning in *Arabidopsis thaliana* . Development 131, 657–668. 14711878 10.1242/dev.00963

[CIT0019] Hedden P 2003 The genes of the Green Revolution. Trends in Genetics 19, 5–9. 12493241 10.1016/s0168-9525(02)00009-4

[CIT0020] Hedden P Phillips AL 2000 Gibberellin metabolism: new insights revealed by the genes. Trends in Plant Science 5, 523–530. 11120474 10.1016/s1360-1385(00)01790-8

[CIT0021] Helliwell CA Chandler PM Poole A Dennis ES Peacock WJ 2001 The CYP88A cytochrome P450, ent-kaurenoic acid oxidase, catalyzes three steps of the gibberellin biosynthesis pathway. Proceedings of the National Academy of Sciences, USA 98, 2065–2070. 10.1073/pnas.041588998 PMC29382 11172076

[CIT0022] Ikeda M Mitsuda N Ohme-Takagi M 2009 Arabidopsis WUSCHEL is a bifunctional transcription factor that acts as a repressor in stem cell regulation and as an activator in floral patterning. Plant Cell 21, 3493–3505. 19897670 10.1105/tpc.109.069997 PMC2798335

[CIT0023] Ishiwata A Ozawa M Nagasaki H 2013 Two *WUSCHEL-related homeobox genes, narrow leaf2* and *narrow leaf3*, control leaf width in rice. Plant and Cell Physiology 54, 779–792. 23420902 10.1093/pcp/pct032

[CIT0024] Itoh H Tatsumi T Sakamoto T Otomo K Toyomasu T Kitano H Ashikari M Ichihara S Matsuoka M 2004 A rice semi-dwarf gene, *Tan-Ginbozu* (*D35*), encodes the gibberellin biosynthesis enzyme, *ent*-kaurene oxidase. Plant Molecular Biology 54, 533–547. 15316288 10.1023/B:PLAN.0000038261.21060.47

[CIT0025] Itoh H Ueguchi-Tanaka M Sato Y Ashikari M Matsuoka M 2002 The gibberellin signaling pathway is regulated by the appearance and disappearance of SLENDER RICE1 in nuclei. The Plant Cell 14, 57–70. 11826299 10.1105/tpc.010319 PMC150551

[CIT0026] Itoh H Ueguchi-Tanaka M Sentoku N Kitano H Matsuoka M Kobayashi M 2001 Cloning and functional analysis of two gibberellin 3 beta -hydroxylase genes that are differently expressed during the growth of rice. Proceedings of the National Academy of Sciences, USA 98, 8909–8914. 10.1073/pnas.141239398 PMC37534 11438692

[CIT0027] Jefferson RA Kavanagh TA Bevan MW 1987 GUS fusions: beta-glucuronidase as a sensitive and versatile gene fusion marker in higher plants. The EMBO Journal 6, 3901–3907. 3327686 10.1002/j.1460-2075.1987.tb02730.x PMC553867

[CIT0028] Jeon JS Lee S Jung KH 2000 T-DNA insertional mutagenesis for functional genomics in rice. The Plant Journal 22, 561–570. 10886776 10.1046/j.1365-313x.2000.00767.x

[CIT0029] Kamiya N Nagasaki H Morikami A Sato Y Matsuoka M 2003 Isolation and characterization of a rice *WUSCHEL*-type homeobox gene that is specifically expressed in the central cells of a quiescent center in the root apical meristem. The Plant Journal 35, 429–441. 12904206 10.1046/j.1365-313x.2003.01816.x

[CIT0030] Kaneko M Itoh H Inukai Y Sakamoto T Ueguchi-Tanaka M Ashikari M Matsuoka M 2003 Where do gibberellin biosynthesis and gibberellin signaling occur in rice plants? The Plant Journal 35, 104–115. 12834406 10.1046/j.1365-313x.2003.01780.x

[CIT0031] Khush GS 1999 Green revolution: preparing for the 21st century. Genome 42, 646–655. 10464789

[CIT0032] Kobayashi MYI Murofushi N Ota Y Takahashi N 1988 Fluctuation and localization of endogenous gibberellins in rice. Agricultural and Biological Chemistry 52, 1189–1104.

[CIT0033] Lee IJ Foster KR Morgan PW 1998 Photoperiod control of gibberellin levels and flowering in sorghum. Plant Physiology 116, 1003–1011. 9501132 10.1104/pp.116.3.1003 PMC35069

[CIT0034] Li J Jiang J Qian Q 2011 Mutation of rice *BC12/GDD1*, which encodes a kinesin-like protein that binds to a GA biosynthesis gene promoter, leads to dwarfism with impaired cell elongation. The Plant Cell 23, 628–640. 21325138 10.1105/tpc.110.081901 PMC3077781

[CIT0035] Livak KJ Schmittgen TD 2001 Analysis of relative gene expression data using real-time quantitative PCR and the 2-^∆∆C^T method. Methods 25, 402–408. 11846609 10.1006/meth.2001.1262

[CIT0036] Lohmann JU Hong RL Hobe M Busch MA Parcy F Simon R Weigel D 2001 A molecular link between stem cell regulation and floral patterning in Arabidopsis. Cell 105, 793–803. 11440721 10.1016/s0092-8674(01)00384-1

[CIT0037] Matsumoto N Okada K 2001 A homeobox gene, *PRESSED FLOWER*, regulates lateral axis-dependent development of Arabidopsis flowers. Genes & Development 15, 3355–3364. 11751640 10.1101/gad.931001 PMC312850

[CIT0038] Matsuo T Futsuhara Y. Kikuchi F. Yamaguchi H 1997 Science of the rice plant . Tokyo, Japan: Nobunkyo p. 302–303.

[CIT0039] Nardmann J Ji J Werr W Scanlon MJ 2004 The maize duplicate genes *narrow sheath1* and *narrow sheath2* encode a conserved homeobox gene function in a lateral domain of shoot apical meristems. Development 131, 2827–2839. 15169755 10.1242/dev.01164

[CIT0040] Olszewski N Sun TP Gubler F 2002 Gibberellin signaling: biosynthesis, catabolism, and response pathways. The Plant Cell 14 Suppl, S61–80. 12045270 10.1105/tpc.010476 PMC151248

[CIT0041] Sakamoto T Matsuoka M 2004 Generating high-yielding varieties by genetic manipulation of plant architecture. Current Opinion in Biotechnology 15, 144–147. 15081053 10.1016/j.copbio.2004.02.003

[CIT0042] Sakamoto T Miura K Itoh H 2004 An overview of gibberellin metabolism enzyme genes and their related mutants in rice. Plant Physiology 134, 1642–1653. 15075394 10.1104/pp.103.033696 PMC419838

[CIT0043] Spielmeyer W Ellis MH Chandler PM 2002 Semidwarf (*sd-1*), “green revolution” rice, contains a defective gibberellin 20-oxidase gene. Proceedings of the National Academy of Sciences, USA 99, 9043–9048. 10.1073/pnas.132266399 PMC124420 12077303

[CIT0044] Swain SM Singh DP 2005 Tall tales from sly dwarves: novel functions of gibberellins in plant development. Trends in Plant Science 10, 123–129. 15749470 10.1016/j.tplants.2005.01.007

[CIT0045] Ueguchi-Tanaka M Fujisawa Y Kobayashi M Ashikari M Iwasaki Y Kitano H Matsuoka M 2000 Rice dwarf mutant *d1*, which is defective in the alpha subunit of the heterotrimeric G protein, affects gibberellin signal transduction. Proceedings of the National Academy of Sciences, USA 97, 11638–11643. 10.1073/pnas.97.21.11638 PMC17253 11027362

[CIT0046] Wang W Li G Zhao J Chu H Lin W Zhang D Wang Z Liang W 2014 DWARF TILLER1, a WUSCHEL-related homeobox transcription factor, is required for tiller growth in rice. PLoS Genetics 10, e1004154. 24625559 10.1371/journal.pgen.1004154 PMC3952828

[CIT0047] Yamaguchi S 2008 Gibberellin metabolism and its regulation. Annual Review of Plant Biology 59, 225–251. 10.1146/annurev.arplant.59.032607.092804 18173378

[CIT0048] Yoo SC Cho SH Paek NC 2013 Rice WUSCHEL-related homeobox 3A (OsWOX3A) modulates auxin-transport gene expression in lateral root and root hair development. Plant Signaling & Behavior 8, e25929. 10.4161/psb.25929 PMC4091085 24002214

[CIT0049] Yoo SC Cho SH Sugimoto H Li J Kusumi K Koh HJ Iba K Paek NC 2009 Rice *virescent3* and *stripe1* encoding the large and small subunits of ribonucleotide reductase are required for chloroplast biogenesis during early leaf development. Plant Physiology 150, 388–401. 19297585 10.1104/pp.109.136648 PMC2675711

[CIT0050] Zhang J Liu X Li S Cheng Z Li C 2014 The rice semi-dwarf mutant *sd37*, caused by a mutation in CYP96B4, plays an important role in the fine-tuning of plant growth. PLoS One 9, e88068. 24498428 10.1371/journal.pone.0088068 PMC3912173

[CIT0051] Zhao Y Hu Y Dai M Huang L Zhou DX 2009 The WUSCHEL-related homeobox gene *WOX11* is required to activate shoot-borne crown root development in rice. The Plant Cell 21, 736–748. 19258439 10.1105/tpc.108.061655 PMC2671696

[CIT0052] Zhu Y Nomura T Xu Y 2006 *ELONGATED UPPERMOST INTERNODE* encodes a cytochrome P450 monooxygenase that epoxidizes gibberellins in a novel deactivation reaction in rice. The Plant Cell 18, 442–456. 16399803 10.1105/tpc.105.038455 PMC1356550

