# Supplementary material for: OsWOX3A is involved in negative feedback regulation of the gibberellic acid biosynthetic pathway in rice (Oryza sativa)
Source: J Exp Bot. 2016 Jan 14;67(6):1677–87. doi: 10.1093/jxb/erv559 (PMC4783357; doi:10.1093/jxb/erv559)
Supplement: Supplementary Data [file supp_67_6_1677__index.html]

OsWOX3A is involved in negative feedback regulation of the gibberellic acid biosynthetic pathway in rice (Oryza sativa) — Supplementary Data 

# OsWOX3A is involved in negative feedback regulation of the gibberellic acid biosynthetic pathway in rice (*Oryza sativa*)

## Supplementary Data

Data files

- supplementary\_figures\_S1\_S6\_table\_S1.pdf - Supplementary Data
