# Supplementary material for: OsWOX3A is involved in negative feedback regulation of the gibberellic acid biosynthetic pathway in rice (Oryza sativa)
Source: J Exp Bot. 2016 Jan 14;67(6):1677–87. doi: 10.1093/jxb/erv559 (PMC4783357; doi:10.1093/jxb/erv559)
Supplement: Supplementary Data [file supp_erv559_supplementary_figures_S1_S6_table_S1.pdf]

Supplementary Information (SI) Appendix

OsWOX3A is involved in negative feedback regulation of the gibberellic acid biosynthetic pathway in rice

Sung-Hwan Cho, Kiyoon Kang, Sang-Hwa Lee, In-Jung Lee and Nam-Chon Paek*

*Author for correspondence: ncpaek@snu.ac.kr

Supplementary Information (SI) contains:

SI Figures 1-6

SI Table 1

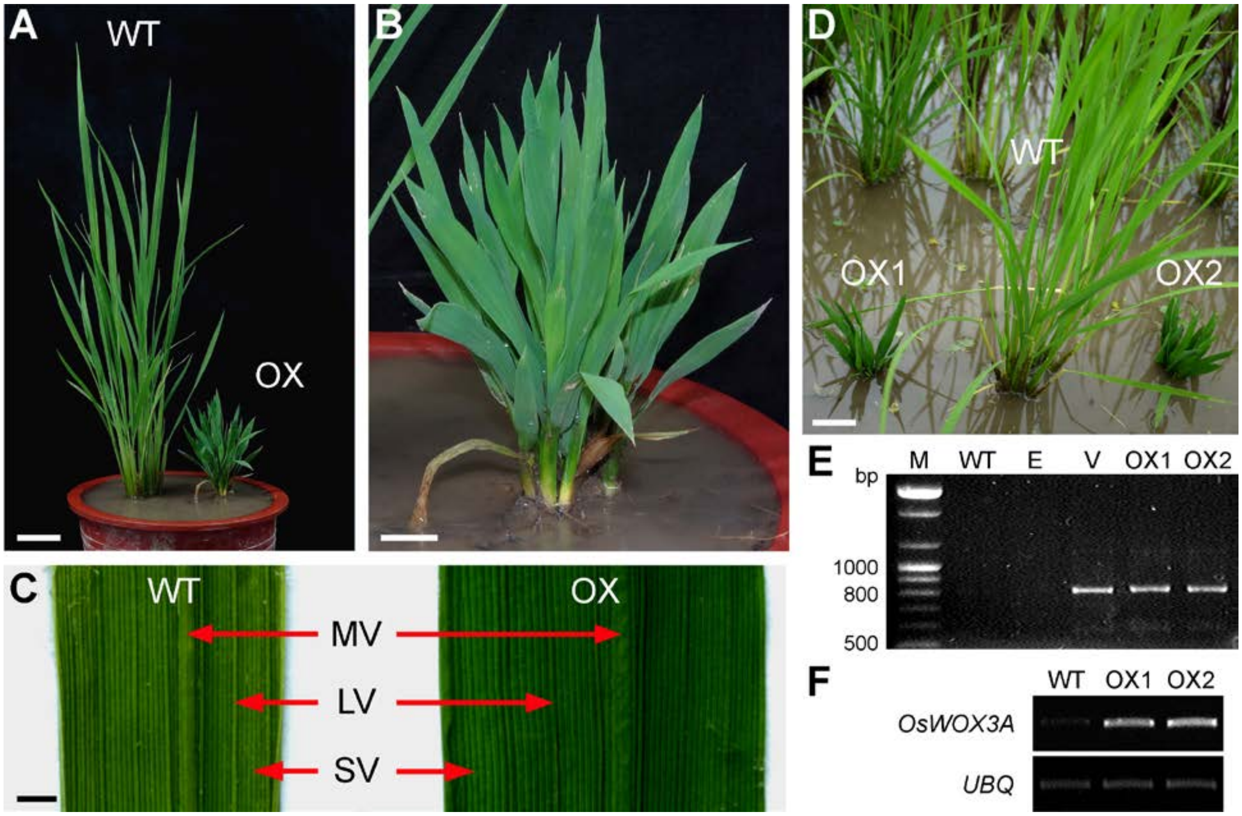

Supplementary Fig. 1. Phenotypic characteristics of *OsWOX3A*-OX (OX) plants.

(A) The 3-month-old wild-type (WT) and OX plants.

(B) Magnified view of OX plant in (A).

(C) Adaxial sides of leaf blades.

(D) The 3-month-old WT and plants from two independent transgenic lines (OX1 and OX2) in the paddy field.

(E) Verification of 2 independent transgenic lines. Genomic DNA was extracted, and an 805-bp PCR product for *OsWOX3A* inside the 2 x 35S promoter region of pMDC32 was amplified.

(F) RT-PCR analysis of *OsWOX3A* overexpression in two transgenic lines (OX1, OX2). MV, midrib vein; LV, large vein; SV, small vein; M, DNA marker; E, empty plasmid of pMDC32 as negative control; V, recombinant plasmid of pMDC32-WOX3A as positive control.

Scale bars = 4 cm (A, D), 2 cm (B), 2 mm (C)

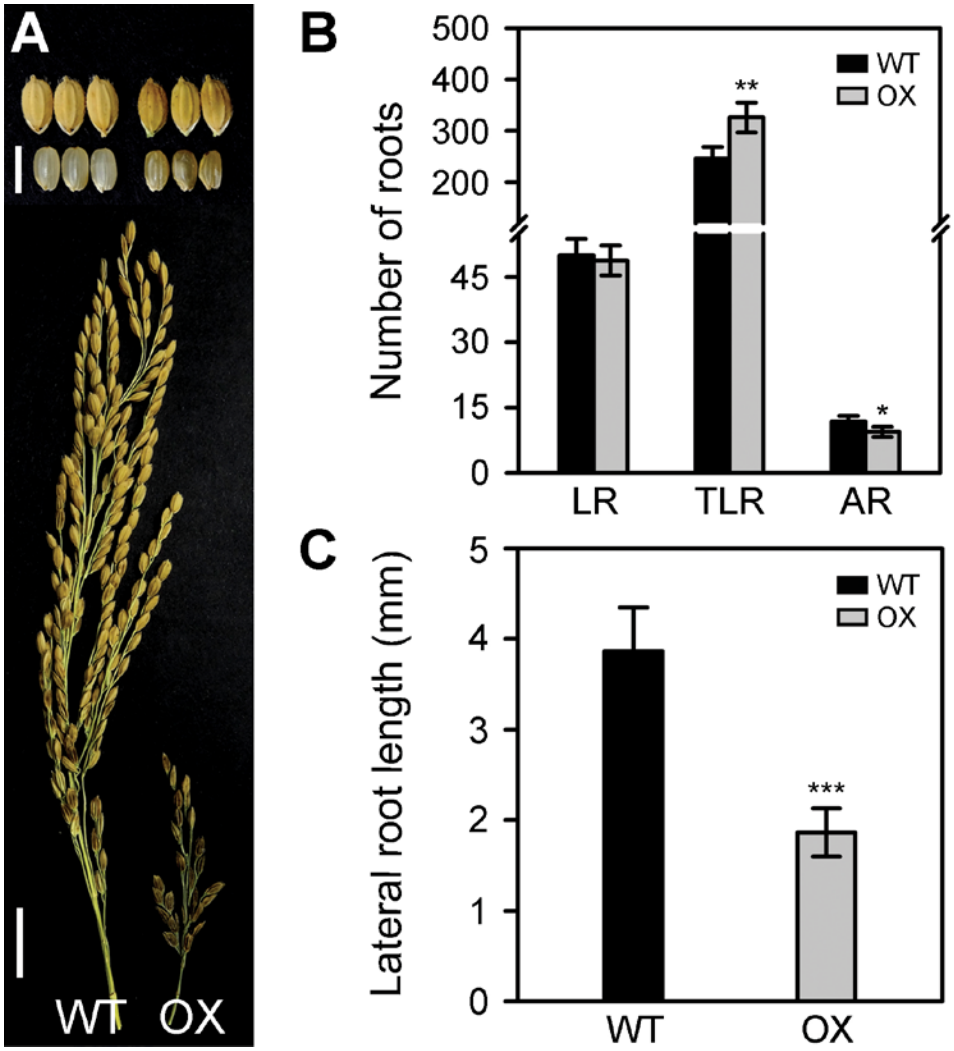

Supplementary Fig. 2. Multiple developmental defects in *OsWOX3A*-OX (OX) plants.

(A) Comparison of spikelet, grain, and panicle shapes between wild-type (WT) and OX plants.

(B) Lateral root (LR), total lateral root (TLR), and adventitious root (AR) number. The LR number within 1.5 cm-length of primary root, TLR number per plant, and AR number per plant were counted with 10-day-old seedlings.

(C) LR lengths in (B). (B, C) Data are means ± SD from 10 plants. Significant difference was determined by Student's *t*-test (*P <0.05, **P<0.01, ***P<0.001).

Scale bars = 5 mm (top, A), 3 cm (bottom, A).

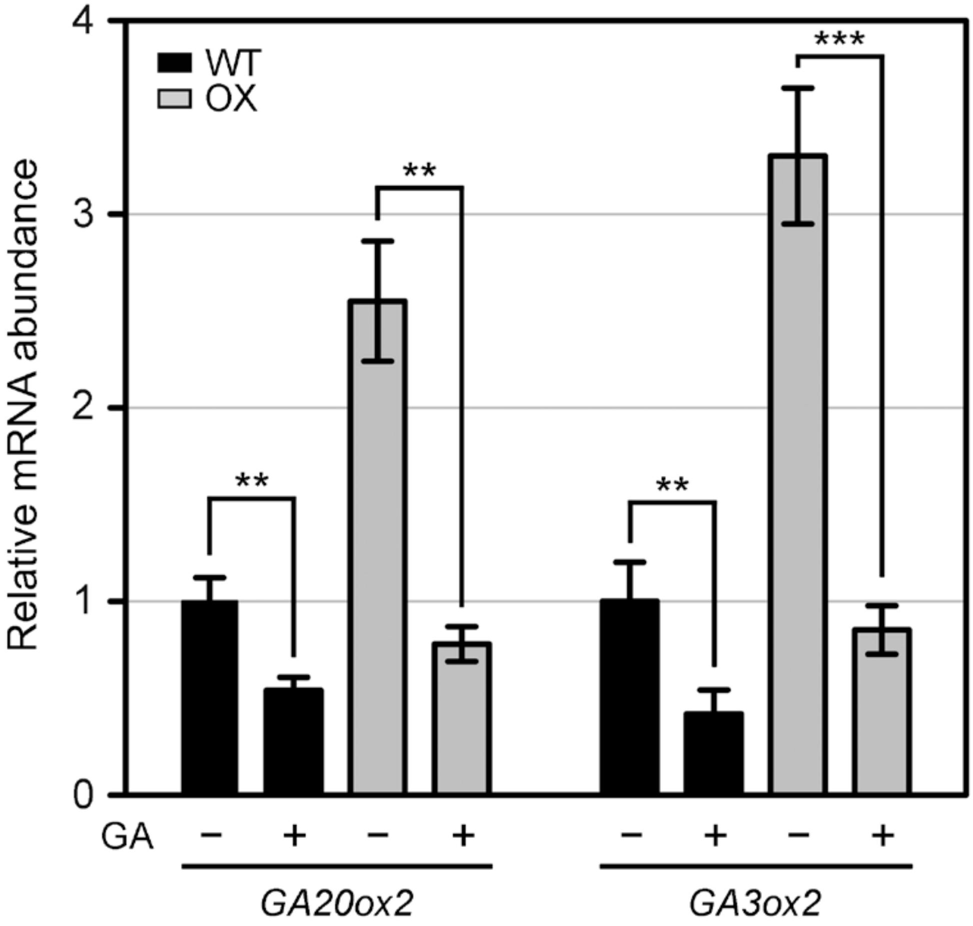

Supplementary Fig. 3. Effect of exogenous GA_3 treatment on the relative expression of *GA20ox2* and *GA3ox2* in 4-week-old wild type (WT) and *OsWOX3A*-OX (OX) plants. By RT-qPCR, relative mRNA levels of each gene are normalized to the mRNA levels of *Ub5* (Os01g0328400). Expression levels of *GA20ox2* and *GA3ox2* after GA treatment (+) are shown relative to those before GA treatment (-) in plants, which is set as 1. Data are means ± SD from three biological repeats. Significant difference was determined by Student's *t*-test (**P < 0.01, ***P < 0.001).

These experiments were repeated more than twice with similar results.

AACAGACAGACAGACTTTATAGCAACATCTAGCAGCAGCAGTAACATCTGTTAGTGTTGACCACCGTCCCCCCCC
CCCCCCCCCCCC**CAAT**GCAACCCCACAGTGACAAAGGCACAACCCTAC**CAAT**GCTTTCTTCTTCCTCCTCCTCTC
TGATTACAGTTACATGAACAG**TTAA**TTTGTTGAGCTTTGCTCACTGCTGCAGGTGATCCCGATCAGTACTAGTAC
TAATTTCTACAGTATGTCTGTCTGACGATGAATGCAGGGTTGCAGACAGACGGACAGCATCTTTTCATCTTCAGT
GTTCTGCACTCTGAATCTCTGACACTGCCTGTGATCCGTGCGAGAGTCTGTCAGTCTCGCTCGTCTGATCAGTGA
TCACCACACTCTG**CAAT**CTGTGGGCTGTGTACCCCATATCTTTTCTAGGATTAGCTTTATGGACTATATATATAT
ATATGTGTGTTTCTCAAAAAGAAATTTCAAGGGGACCCGGCTAGAAAGAAAGAAAGAGATCCTGCCTGCACTAGT
AGTAGTAGCTTTATTATCGTGTTG**CAAT**TGCATCCTCTCTCTCTCTCTCTCTCAACTCTGAACTGATCTTCTC
GAATATCTCCATAATTCAGAATGGAGAGGCTTCAACCAGAGATGATCATGTTTGGTATGGTGTCACAGTTAGTAA
AGTCCTGCAAAAAAAATAAAAAGGGAGAATTCTTTTTTTTTTCAGATTGTAATACATACCTTCTCTATTATGTAA
AAAATGGTAGTATGTTTTACTAATGGACAGCATTATCTTAGGGATGCATGCATGGTTTACTCC**TTAA**AACTGACT
AACTAATCTTATCGATCTTATCTTCTCACTGGGGAATTCTTCTTCTTTTTCTT**TTAA**CTAAACAAGTGGAGAATG
GTTGGATGGTCCAGAAAATCAGAACAGATAGCATGCGCATGC**CAAT**TGATCCTATCCTCATTCTTATCTGTACTT
GCATGGATAGTAACATGTGGAAGAAGAAGGAACTAA**TTAA**CCAGTTGTCCTCC**TTAA**TCATTACTAAATTTAGGC
GAATGCATGCATGCGCTGC**TTAATTAA**GCCAGCCAAAATGGTAAAAGCTTTAGTAC**CAAT**CCACATGAGGTAGTA
ATTAGT**CAAT**CAAACATTCATTCATTTAGGATCCACCATCCACCATCTCGATCAGGAACAAAACTGATGACTACC
ACAGATATATATATATATATATATATATATTAGCATATAGCTCATCTTTTTTTTTACCAGAATATGAAAAATTCAGT
ACAGTCTATACGCAAGTAAAAA**TTAA**AATGTTTTCTTTTATAGCTTTATGATTTTTTTTTACAGAAGAA**CAAT**CT
GAATGATTATTT**TTAA**AAGATATTAGAAGCTTTA**TTAA**ACTCAACGTGATACGAGTTGGATGAAATAGACCGTGC
AACTACTAGCTAGAAGAGATACGTAATATATCCATGTGTGGCCATGTAAATGGAGTCGTTGCTACATACAAA**TTA**
ATTGAGCTAGATACATATGGAGAGCAAAACCATGG**TTAA**TCAGTTTTGCATTTCGATCACTGGAATTGATGGACT
GTGTTGTTA**TTAA**GCTAGGGTACAAGAAAAGCCGGCGATAAATTAGATGGATCGAAACCAAACCACAAGAGTCCA
AAGAAGAAAGTACAAAGGATCTCTGGACATGCTCACGATGAATAATCCCATCGATCGAGCTACTATACATACAAA
ACTCACTACTAGTAA**TTAA**GTGTTGAGGGTTCGAGAACAAA**TTAATTAATCG**CCAAATAATGCATCACAAACATC
ATCAACCTAGTTATCGGTTTCTTTTT**TTAA**ATCGAGAACATCTTGA**TTAA**TTATGTAAAGCCCTTTTTGTCCTCT
CGAAAAAAAAAAAAGCTAAGCACGCATTTTTTTCCGCGTAAA**TTAA**AATTAGAGAAATAAATAAATAAAGGAGGG
AATTGGGCAGTCGCCAGGGTATATATTGAGAGGGAGGAGCTTAGCATTTGGCGACACTCGTCTCTGCGTGCGTTT
CGTTCCTTCGTTGCGTTGCGTTGGTTCATCGATAGATCGTTCTCG**ATG**

Supplemental Fig. 4. Analysis of OsWOX3A-binding motifs in the promoter of *KAO*. The 2-kb promoter region of *KAO* upstream sequences was obtained from the NCBI database. The OsWOX3A-binding sites are shown in red (TTAATCG). The other previously reported binding elements for WOX13 are shown in blue (CAAT) and orange (TTAA). The probe of W3 is shown as underlined sequences (see Fig. 6A). ATG (bold letters) at the end indicates the start codon of *KAO*.

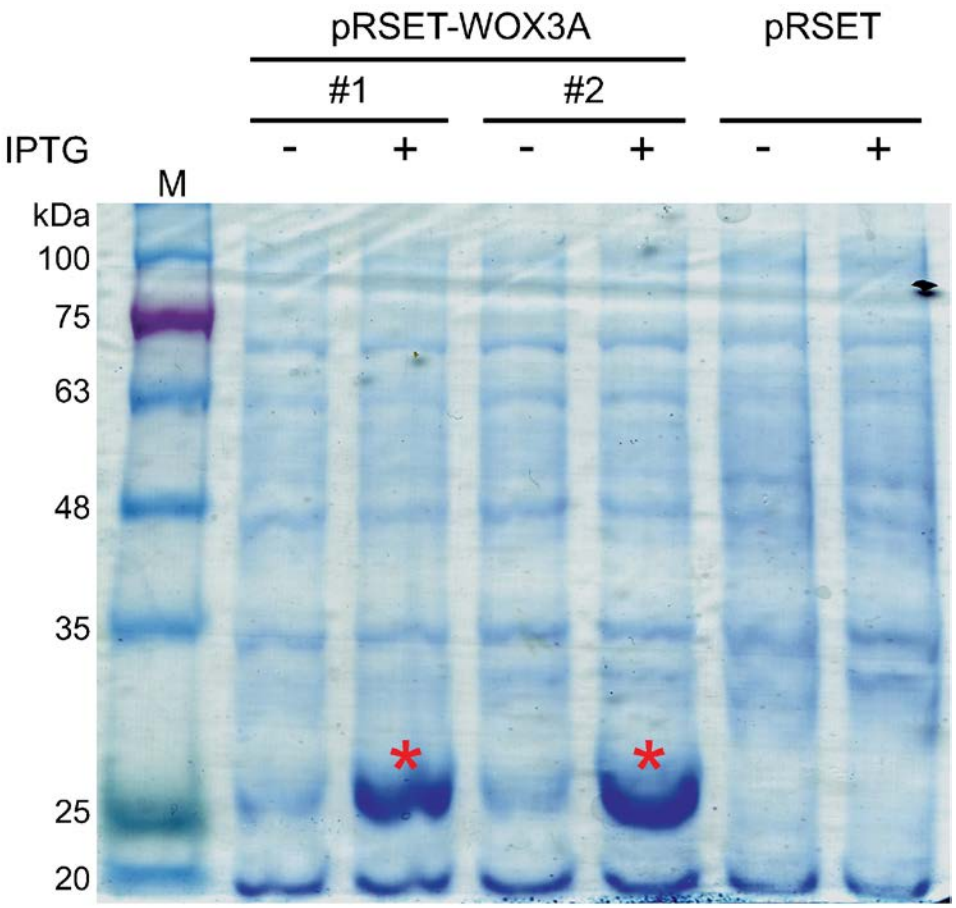

Supplementary Fig. 5. Expression of recombinant OsWOX3A fusion protein in *E*. *coil*. Recombinant His-OsWOX3A fusion protein was produced by BL21 *E*. *coli* after 3 h incubation with 1 mM IPTG. Bacterial pellet was collected, resuspended in 100 µl PBS buffer and subjected to freeze–thaw cycles four times. An equal volume of 2x SDS-polyacrylamide gel electrophoresis sample buffer was added to the supernatant. A total of 25 µl of each sample was loaded on SDS-PAGE and stained with Coomassie brilliant blue. A total of 28 kDa His-OsWOX3A fusion protein including tags was detected after the IPTG induction. M, protein size marker. Red asterisks indicate recombinant His-OsWOX3A fusion protein.

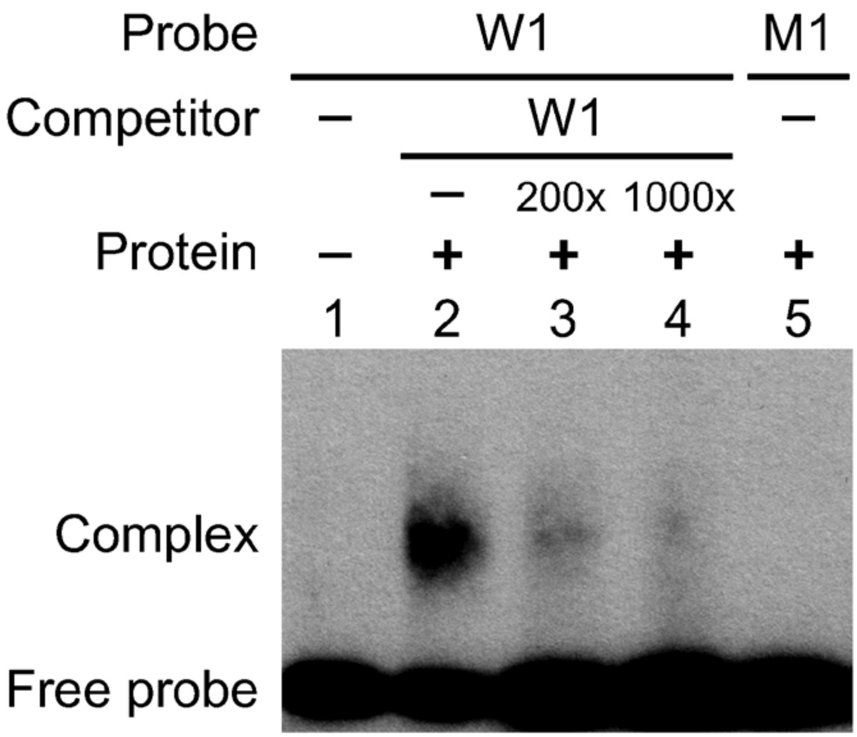

Supplementary Fig. 6. OsWOX3A does not bind to the M1 promoter region of *KAO*. EMSA using the His-OsWOX3A fusion protein with W1 and M1 probes. Oligonucleotides containing W1 (the *KAO* promoter binding site) and M1 (deleted *KAO* promoter binding site version of W1), were used as the biotin-labeled probes. Negative control is indicated in Lane 1. W1 was used as the unlabeled competitor. The (+) presence or (-) absence of His-OsWOX3A fusion protein is indicated.
These experiments were repeated more than three times with similar results

Supplementary Table 1. Primers used in this study. Underlined sequences are restriction enzyme sequences.

Product	Sequence (5′ → 3′)
**Cloning & confirmation of transgenic plants**	
35STC-F	ATTGCCCAGCTATCTGTCACTT
TC-R	ATCGCCATGTCAGCAGCAGTT
OsWOX3A-F	ATGCCTCAGACCCCTTCGACGCGGTGGT
OsWOX3A-R	TTAATTGGTGGAGGTGGAGCAAGAGGAGGA
**RT-qPCR analysis**	
OsWOX3A-sRT-F	GCAACTGCTGCTGACATGGC
OsWOX3A-sRT-R	AAGAGGAGGACTTGGAGCTGC
CPS1-Real-F	GCGTGCATTTTCGAACCAA
CPS1-Real-R	TTGGCCAGCACTGACACTCT
KO1-Real-F	AGTAGCCAAGGAGGCGATGA
KO1-Real-R	CGGCTTATCACAGACAATGCT
KO2-Real-F	TGAAGTAGCCAAGGAGGCGA
KO2-Real-R	CGCTGATTGCGACCATACTTT
KAO-Real-F	CGCAAGAGCAAAGGCTGAG
KAO-Real-R	CCTGTGAGAGGAAGTGCATCTTCT
GA20ox2-F	GGGAGGGTGTACCAGAAGTACTG
GA20ox2-R	GGCTCAGCTCCAGGAGTTCC

GA3ox2-F	TCTTCTCCAAGCTCATGTGGT
GA3ox2-R	AACTCCTCCATCACGTCACAG
GA2ox1-F	TGACGATGATGACAGCGACAA
GA2ox1-R	CCATAGGCATCGTCTGCAATT
GA2ox3-F	TGGTGGCCAACAGCCTAAAG
GA2ox3-R	TGGTGCAATCCTCTGTGCTAAC
PIN1b-F	TTCTGCACATTGCCATTGTT
PIN1b-R	AATGTGATGGGGAGAGCAAT
PIN1c-F	ATCGTGCAGGCAGCGTTGCC
PIN1c-R	ATGTAGTACACCAGCGTGAT
PIN2-F	TGTCAGATGCAGGGCTAGGAA
PIN2-R	TGCCACAAGAAATGATCTTTGG
Actin-F	TGCTATGTACGTCGCCATCCAG
Actin-R	AATGAGTAACCACGCTCCGTCA
Ub5-F	GTCTGATCTTCGCTGGCAAGCAGC
Ub5-R	GCATACTGCTGTCCCACAGGAAACTG
**Yeast one-hybrid assay**	
CPS1_pro-F	GAATTCTTTATGTCAAATCTTAATGCAA
CPS1_pro-R	GTCGACTATAGCCCACTTCCCTCGCCAT
KO2_pro-F	AAGCTTATTATTAAAAATTGATCGGGCCTT
KO2_pro-R	CTCGAGTTAATCTTCTTTCTTCTTTCAAACA

pro-KAO-W2-F	GGTACCAAAAATGGTAGTATGTTTTA
pro-KAO-W2-R	GTCGACAACCGATAACTAGGTTGATG
pro-KAO-M2-F	GGTACCAAAAATGGTAGTATGTTTTA
pro-KAO-M2-R	GTCGACAACCGATAACTAGGTTGATGATGTTTGTGATGCATTATTTGGTTAATTTGTTCTCGAAC
pro-KAO-W4-F	GGTACCTTACGGTCACTTGCATGCCTT
pro-KAO-W4-R	GTCGACTAAAACATACTACCATTTTTTACAT
GA3ox2_pro-F	AAGCTTAGTGTGTTGCTCTCTGGTCAT
GA3ox2_pro-R	GAGCTCATGACCAGAGAGCAACACACT
GA20ox2_pro-F	AAGCTTTAGTTTAGGCGATCGCTTAGAT
GA20ox2_pro-R	CTCGAGTGTGAGTGTGAGTGTGTGTGTG
GA2ox1_pro-F	GAATTCACAACTAGATCGTGTAGCGATTAA
GA2ox1_pro-R	CTCGAGCATGTGTCAAGCAAGCCACAAATCT
GA2ox3_pro-F	AAGCTTTGAATGCTGAAATTCACCATACGT
GA2ox3_pro-R	GTCGACCTTTATCTGGTGGTGGCAATGGA
OsWOX3A-BamH1-F	GGATCCATGCCTCAGACCCCTTCGACGCGGTGGT
OsWOX3A-Sal1-R	GTCGACTTAATTGGTGGAGGTGGAGCAAGAGGAGGA
**EMSA**	
KO2-1-F	ATTATTAAAAATTGATCGGGCCTT
KO2-1-R	CTCAAAGAATTTACGTCAGTT
KO2-3-F	GAGGTAGCTGCTACACGTGTT
KO2-3-R	TCAAACACACTAGAATAATAAGT

KO2-1-DEL-F	ATTAAATTGATCGGGCCAAAACTATCATGG
KO2-1-DEL-R	CTCAAAGAATTTACGTCAGTT
KAO-W1-F	TACAAAACTCACTACTAGTA
KAO-W1-R	AACCGATAACTAGGTTGATG
KAO-M1-F	TACAAAACTCACTACTAGTA
KAO-M1-R	GATGATGTTTGTGATGCATTATTTGGTTAATTTGTT
KAO-W3-F	AAGCCAGCCAAAATGGTAAAA
KAO-W3-R	AAACTGATGACTACCACAGA
OsWOX3A-BamH1-F	GGATCCATGCCTCAGACCCCTTCGACGCGGTGGT
OsWOX3A-EcoR1-R	GAATTCTTAATTGGTGGAGGTGGAGCAAGAGGAGGA
